# Supplementary material for: Is adaptation limited by mutation? A timescale-dependent effect of genetic diversity on the adaptive substitution rate in animals
Source: PLoS Genet. 2020 Apr 6;16(4):e1008668. doi: 10.1371/journal.pgen.1008668 (PMC7162527; doi:10.1371/journal.pgen.1008668)
Supplement: S1 Text — (PDF) [file pgen.1008668.s001.pdf]

One of the two estimates of the adaptive rate at group level used in this study, $\omega_{a[A]}$, was obtained by calculating the across-species arithmetic mean of ω_{na} within a group, then subtracting this average from ω. Here we show that $\omega_{a[A]}$ is an unbiased estimate of the adaptive rate with fluctuating population size if the pace of fluctuations is sufficiently slow. We justify this strategy by making the hypothesis that the present variation in population sizes between closely related species represents well the possible range of population size fluctuations that one population experienced during the time period of its divergence with its sister species. Under our model, ω_{na} for an individual species is

(1) $$\omega_{na} = (\widehat{D_N^{na}}/L_N)/(D_S/L_S),$$

where $\widehat{D_N^{na}}$ is the expected number of non-synonymous substitutions:

(2) $$\widehat{D_N^{na}} = 2L_N N_e t\mu \int_{-\infty}^{0} \phi(s) f(N_e, s) ds$$

where L_N is the number of non-synonymous sites, t is the divergence time, $\phi(s)$ is the fixation probability of a mutation with a selection coefficient s, and $f(Ne, s)$ is the DFE, in a population of size N_e.

Then, if we have sampled n closely related species, and we call N_{e1}, N_{e2}, N_{e3}, … N_{en} their respective present effective population size, then, making the hypothesis that the shape of the DFE and the mutation rate μ remains constant in time, one can express the expected number of non-synonymous substitutions, $\widehat{D_N^{na}}$, as :

(3) $$\widehat{D_N^{na}} = \frac{t}{n}\sum_{i}^{n} 2L_N N_{ei}\mu \int_{-\infty}^{0} \phi(s) f(N_{ei,s}) ds$$

Equation (3) is equivalent to assuming that, as the considered species were diverging, they have randomly fluctuated between the n regimes of selection/drift we currently observe, spending the same amount of time in the n regimes. Under this assumption, the group level $\widehat{D_N^{na}}$ is simply the arithmetic mean of $\widehat{D_N^{na}}$ estimated across individual species. Then, using L_N, L_S and D_S of the total subtree of the considered species, we can use the arithmetic mean of ω_{na} across individual species, $\omega_{na[A]}$, as representative of the average non-adaptive selective regime during their divergence. Subtracting $\omega_{na[A]}$ from the dN/dS ratio estimated using all branches of the tree, we obtain an estimate of the adaptive substitution rate for the whole group, $\omega_{a[A]}$.

S1 Text: Rationale of the estimation of the per group adaptive substitution rate “A”.
