# Supplementary material for: Is adaptation limited by mutation? A timescale-dependent effect of genetic diversity on the adaptive substitution rate in animals
Source: PLoS Genet. 2020 Apr 6;16(4):e1008668. doi: 10.1371/journal.pgen.1008668 (PMC7162527; doi:10.1371/journal.pgen.1008668)
Supplement: S1 Table — (DOC) [file pgen.1008668.s002.doc]

| **Species** | **Bioproject** | **data_type** | **#individuals** | **publication** | **Reference genome** |
| --- | --- | --- | --- | --- | --- |
| *Gorilla gorilla* | PRJNA189439 | Genome | 20 | Prado-Martinez et al. 2013 | Ensembl (release 89) |
| *Homo sapiens* | PRJEB8350 | Exome | 19 | Teixeira et al. 2015 | Ensembl (release 89) |
| *Pan troglodytes* | PRJEB8350 | Exome | 20 | Teixeira et al. 2015 | Ensembl (release 89) |
| *Papio anubis* | PRJNA54005 | Genome | 5 | unpublished baboon genome project | Ensembl (release 89) |
| *Pongo abelii* | PRJNA189439 and PRJEB1675 | Genome | 10 | Prado-Martinez et al. 2013 | Ensembl (release 89) |
| *Macaca mulatta* | PRJNA251548 | Exome | 20 | Xue et al. 2016 | Ensembl (release 89) |
| *Meleagris gallopavo* | PRJNA271731 | RNA-seq | 10 | Wright et al. 2015 | NA |
| *Phasianus colchicus* | PRJNA271731 | RNA-seq | 11 | Wright et al. 2015 | NA |
| *Pavo cristatus* | PRJNA271731 | RNA-seq | 10 | Wright et al. 2015 | NA |
| *Numida meleagris* | PRJNA271731 | RNA-seq | 7 | Wright et al. 2015 | NA |
| *Anas platyrhynchos* | PRJNA271731 | RNA-seq | 10 | Wright et al. 2015 | NA |
| *Anser cygnoides* | PRJNA271731 | RNA-seq | 10 | Wright et al. 2015 | NA |
| *Ficedula albicollis* | PRJEB2984 | Genome | 20 | Ellegren et al. 2012 | NCBI FicAlb1.5 |
| *Geospiza difficilis* | PRJNA263122 | Genome | 8 | Lamichhaney et al. 2015 | NCBI Geofor1.0 |
| *Parus major* | PRJNA381923 | Genome | 10 | Corcoran et al. 2017 | //ftp.ncbi.nlm.nih.gov/genomes/all/GCF/001/522/545/GCF_001522545.2_Parus_major1.1/GCF_001522545.2_Parus_major1.1_genomic.gff.gz |
| *Corvus sp.* | PRJEB9057 | Genome | 10 | Vijay et al. 2017 | //ftp.ncbi.nlm.nih.gov/genomes/all/GCF/000/738/735/GCF_000738735.2_ASM73873v2/GCF_000738735.2_ASM73873v2_genomic.gff.gz |
| *Taniopygia guttata* | PRJEB10586 | Genome | 20 | Singhal et al. 2016 | //ftp.ncbi.nlm.nih.gov/genomes/all/GCF/000/151/805/GCF_000151805.1_Taeniopygia_guttata-3.2.4/GCF_000151805.1_Taeniopygia_guttata-3.2.4_genomic.gff.gz |
| *Maniola jurtina* | PRJNA530965 | target capture | 20 | newly generated | NA |
| *Melanargia galathea* | PRJNA530965 | target capture | 10 | newly generated | NA |
| *Aphantopus hyperantus* | PRJNA530965 | target capture | 7 | newly generated | NA |
| *Pyronia tithonus* | PRJNA530965 | target capture | 7 | newly generated | NA |
| *Pyronia bathseba* | PRJNA530965 | target capture | 8 | newly generated | NA |
| *Formica sanguinea* | PRJNA530965 | target capture | 10 | newly generated | NA |
| *Formica cunicularia* | PRJNA530965 | target capture | 6 | newly generated | NA |
| *Formica pratensis* | PRJNA530965 | target capture | 8 | newly generated | NA |
| *Formica fusca* | PRJNA530965 | target capture | 8 | newly generated | NA |
| *Allolobophora chlorotica L1* | PRJNA530965 | target capture | 19 | newly generated | NA |
| *Allolobophora chlorotica L2* | PRJNA530965 | target capture | 8 | newly generated | NA |
| *Allolobophora chlorotica L4* | PRJNA530965 | target capture | 9 | newly generated | NA |
| *Aporrectodea icterica* | PRJNA530965 | target capture | 10 | newly generated | NA |
| *lumbricus terrestris* | PRJNA530965 | target capture | 9 | newly generated | NA |
| *Lineus lacteus* | PRJNA530965 | target capture | 9 | newly generated | NA |
| *Lineus sanguineus* | PRJNA530965 | target capture | 9 | newly generated | NA |
| *Lineus longissimus* | PRJNA530965 | target capture | 6 | newly generated | NA |
| *Lineus ruber* | PRJNA530965 | target capture | 8 | newly generated | NA |
| *Mytilus galloprovincialis* | PRJNA530965 | target capture | 9 | newly generated | NA |
| *Mytilus californianus* | PRJNA530965 | target capture | 16 | newly generated | NA |
| *Mytilus edulis* | PRJNA530965 | target capture | 10 | newly generated | NA |
| *Mytilus trossulus* | PRJNA530965 | target capture | 10 | newly generated | NA |
| *Drosophila melanogaster* | SRP006733 | Genome | 10 | Pool et al. 2012 | //ftp.ncbi.nlm.nih.gov/genomes/all/GCF/000/001/215/GCF_000001215.4_Release_6_plus_ISO1_MT/GCF_000001215.4_Release_6_plus_ISO1_MT_genomic.fna.gz |
| *Drosophila sechellia* | PRJNA395473 | Genome | 8 | Schrider et al. 2018 | ftp://ftp.ncbi.nlm.nih.gov/genomes/all/GCF/000/005/215/GCF_000005215.3_dsec_caf1/GCF_000005215.3_dsec_caf1_genomic.fna.gz |
| *Drosophila simulans* | PRJNA215932 | Genome | 10 | Rogers et al. 2014 | ftp://ftp.ncbi.nlm.nih.gov/genomes/all/GCF/000/754/195/GCF_000754195.2_ASM75419v2/GCF_000754195.2_ASM75419v2_genomic.fna.gz |
| *Drosophila santomea* | PRJNA395473 | Genome | 17 | Turissini & Matute 2017 | ftp://ftp.ncbi.nlm.nih.gov/genomes/all/GCF/000/005/975/GCF_000005975.2_dyak_caf1/GCF_000005975.2_dyak_caf1_genomic.fna.gz |
| *Drosophila yakuba* | PRJNA395473 | Genome | 20 | Turissini & Matute 2017 | ftp://ftp.ncbi.nlm.nih.gov/genomes/all/GCF/000/005/975/GCF_000005975.2_dyak_caf1/GCF_000005975.2_dyak_caf1_genomic.fna.gz |
| *Drosophila teissieri* | PRJNA395473 | Genome | 11 | Turissini & Matute 2017 | ftp://ftp.ncbi.nlm.nih.gov/genomes/all/GCF/000/005/975/GCF_000005975.2_dyak_caf1/GCF_000005975.2_dyak_caf1_genomic.fna.gz |
| *Mus musculus castaneus* | PRJEB2176 | Genome | 10 | Harr et al. 2016 | ftp://ftp.ncbi.nlm.nih.gov/genomes/all/GCF/000/001/635/GCF_000001635.26_GRCm38.p6/GCF_000001635.26_GRCm38.p6_genomic.fna.gz |
| *Mus spretus* | PRJEB11742 | Genome | 8 | Harr et al. 2016 | ftp://ftp.ncbi.nlm.nih.gov/genomes/all/GCA/001/624/865/GCA_001624865.1_SPRET_EiJ_v1/GCA_001624865.1_SPRET_EiJ_v1_genomic.fna.gz |
| *Rattus norvegicus* | PRJEB2922 | Genome | 12 | Deinum et al. 2015 | ftp://ftp.ncbi.nlm.nih.gov/genomes/all/GCF/000/001/895/GCF_000001895.5_Rnor_6.0/GCF_000001895.5_Rnor_6.0_genomic.fna.gz |
| *Microtus ochrogaster* | PRJNA428754 | RNA-seq | 18 | NA | NA |
| *Microtus arvalis* | PRJNA249058 | RNA-seq | 7 | Romiguier et al. 2014 | NA |

**References :**

1. Corcoran P, Gossmann TI, Barton HJ, Great Tit HapMap Consortium, Slate J, Zeng K. 2017. Determinants of the efficacy of natural selection on coding and noncoding variability in two passerine species. Genome biology and evolution. 9:2987-3007.
2. Deinum EE, Halligan DL, Ness RW, Zhang YH, Cong L, Zhang JX, Keightley PD. 2015. Recent evolution in Rattus norvegicus is shaped by declining effective population size. Molecular biology and evolution. 32:2547-58.
3. Ellegren H, Smeds L, Burri R, Olason PI, Backström N, Kawakami T, Künstner A, Mäkinen H, Nadachowska-Brzyska K, Qvarnström A, et al. 2012. The genomic landscape of species divergence in *Ficedula* flycatchers. Nature 491:756–760.
4. Harr B, Karakoc E, Neme R, Teschke M, Pfeifle C, Pezer Ž, Babiker H, Linnenbrink M, Montero I, Scavetta R, Abai MR. 2016. Genomic resources for wild populations of the house mouse, Mus musculus and its close relative Mus spretus. Scientific data. 3:160075.
5. Kim J-M, Santure AW, Barton HJ, Quinn JL, Cole EF, Great Tit HapMap Consortium, Visser ME, Sheldon BC, Groenen MAM, van Oers K, et al. 2018. A high-density SNP chip for genotyping great tit ( *Parus major* ) populations and its application to studying the genetic architecture of exploration behaviour. Molecular Ecology Resources 18:877–891.
6. Lamichhaney S, Berglund J, Almén MS, Maqbool K, Grabherr M, Martinez-Barrio A, Promerová M, Rubin C-J, Wang C, Zamani N, et al. 2015. Evolution of Darwin’s finches and their beaks revealed by genome sequencing. Nature 518:371–375.
7. Pool JE, Corbett-Detig RB, Sugino RP, Stevens KA, Cardeno CM, Crepeau MW, Duchen P, Emerson JJ, Saelao P, Begun DJ, Langley CH. 2012. Population genomics of sub-Saharan Drosophila melanogaster: African diversity and non-African admixture. PLoS genetics. 8(12):e1003080.
8. Prado-Martinez J, Sudmant PH, Kidd JM, Li H, Kelley JL, Lorente-Galdos B, Veeramah KR, Woerner AE, O’Connor TD, Santpere G. 2013. Great ape genetic diversity and population history. Nature 499:471.
9. Teixeira JC, de Filippo C, Weihmann A, Meneu JR, Racimo F, Dannemann M, Nickel B, Fischer A, Halbwax M, Andre C. 2015. Long-term balancing selection in LAD1 maintains a missense trans-species polymorphism in humans, chimpanzees, and bonobos. Molecular biology and evolution 32:1186–1196.
10. Rogers RL, Cridland JM, Shao L, Hu TT, Andolfatto P, Thornton KR. 2014. Landscape of standing variation for tandem duplications in Drosophila yakuba and Drosophila simulans. Molecumar Biologu and Evolution. 31:1750– 1766.
11. Romiguier J, Gayral P, Ballenghien M, Bernard A, Cahais V, Chenuil A, Chiari Y, Dernat R, Duret L, Faivre N, Loire E. et al. 2014. Comparative population genomics in animals uncovers the determinants of genetic diversity. Nature 515:261.
12. Schrider DR, Ayroles J, Matute DR, Kern AD. 2018. Supervised machine learning reveals introgressed loci in the genomes of Drosophila simulans and D. sechellia. PLoS genetics. 4(4):e1007341.
13. Turissini DA, Matute DR. 2017. Fine scale mapping of genomic introgressions within the Drosophila yakuba clade. PLoS genetics. 13(9):e1006971.
14. Vijay N, Weissensteiner M, Burri R, Kawakami T, Ellegren H, Wolf JBW. 2017. Genome-wide signatures of genetic variation within and between populations - a comparative perspective. Available from: http://biorxiv.org/lookup/doi/10.1101/104604
15. Wright AE, Harrison PW, Zimmer F, Montgomery SH, Pointer MA, Mank JE. 2015. Variation in promiscuity and sexual selection drives avian rate of Faster‐Z evolution. Molecular ecology 24:1218–1235.

**S1 Table : Details of the species used in this study and numbers of individuals for each species.**
