# Supplementary material for: Is adaptation limited by mutation? A timescale-dependent effect of genetic diversity on the adaptive substitution rate in animals
Source: PLoS Genet. 2020 Apr 6;16(4):e1008668. doi: 10.1371/journal.pgen.1008668 (PMC7162527; doi:10.1371/journal.pgen.1008668)
Supplement: S3 Table — SNPs counts are not integers because they corresponds to SNPs that are present in our SFS, where we chose a sample size (i.e. the number of categories of the SFS) lower that 2*n, where n is the number of individuals. This is to compensate the uneven coverage between individuals that results in some sites in some individuals not to be genotyped. We chose sample sizes that maximize the number of SNPs in each SFS. (PDF) [file pgen.1008668.s004.pdf]

species	Number of indivduals	Chosen sample size	# non-synonymous SNPs	# synonymous SNPs	# SNPs total	#GC-cons, Non-synonymous SNPs	#GC-cons, Synonymous SNPs	#GC-cons, SNPs total
F. fusca	8	10	4278	6578	10856	270	166	436
F. sanguinea	10	16	3242	5343	8585	363	224	587
F. cunicularia	6	8	4035	6355	10390	212	121	333
F. pratensis	8	12	1773	2235	4008	191	87	278
M. galathea	10	10	1190	3309	4499	621	346	966
M. jurtina	20	32	7310	15926	23235	1863	2036	3898
A. hyperanthus	7	8	1441	2385	3826	734	350	1084
P. tithonus	7	10	1534	2372	3906	369	278	647
P. bathseba	8	10	1611	2676	4287	395	315	710
M. californianus	16	24	6370	13436	19806	1467	2209	3675
M. trossulus	10	14	6329	18994	25323	1665	3382	5047
M. galloprovincialis	9	12	3298	9089	12387	839	1665	2504
M. edulis	10	12	5497	15987	21485	1326	2590	3915
A. chlorotica L1	19	26	1751	3094	4845	472	442	914
A. chlorotica L2	8	8	350	554	904	69	56	126
A. chlorotica L4	9	12	3562	7895	11457	701	808	1508
A. icterica	10	12	1657	3778	5435	360	387	747
L. terrestris	9	8	238	940	1178	38	77	115
L. lacteus	9	12	5896	20421	26317	1292	2495	3788
L. longissimus	6	8	55	99	154	11	7	18
L. sanguineus	9	10	954	3248	4202	192	397	589
L. ruber	8	12	1028	1691	2718	196	168	364
H. sapiens	19	28	2281	2812	5093	264	123	387
P. troglodytes	20	30	4744	6558	11302	559	266	825
G. gorilla	20	30	3649	4842	8491	370	180	550
P. anubis	5	8	2038	4006	6044	242	164	407
P. abelii	10	16	5687	9300	14987	594	360	954
M. mulatta	19	28	5257	9976	15232	608	560	1169
A. pltyrhynchos	10	16	3796	13089	16884	206	438	645
A. cygnoides	10	14	1296	4824	6119	103	127	230
M. gallopavo	10	10	1005	3791	4795	61	52	113
N. meleagris	10	16	1319	6241	7560	90	135	224
P. cristatus	10	14	602	2214	2816	48	51	99
P. colchicus	11	14	3051	9703	12754	172	172	343
P. major	10	16	6906	14414	21320	2702	2292	4994
F. albicolis	20	16	16242	20681	36923	2818	2224	5042
Corvus sp.	10	14	817	1355	2172	115	83	197
G. difficilis	8	10	2090	3280	5370	374	232	606
T. guttata	20	16	36318	102249	138567	6588	7775	14363
R. norvegicus	12	18	5023	10224	15246	645	574	1220
M. arvalis	7	10	1783	6351	8134	232	377	609
M. ochrogaster	18	18	2455	5340	7795	448	354	801
M. spretus	8	12	58687	14289	72976	812	921	1733
M. m. castaneus	10	12	5524	17996	23520	778	1068	1846
D. melanogaster	10	16	36213	138306	174518	9574	15862	25436
D. teissieri	11	18	48565	247605	296171	14259	35930	50188
D. santomea	17	28	16104	54878	70982	2374	4142	6516
D. yakuba	20	12	37921	164003	201924	11090	22635	33725
D. simulans	10	16	74864	352964	427828	21397	47094	68490
D. sechellia	8	12	379	591	970	92	61	153

S3 Table : SNPs counts for each species.
