# Supplementary material for: Is adaptation limited by mutation? A timescale-dependent effect of genetic diversity on the adaptive substitution rate in animals
Source: PLoS Genet. 2020 Apr 6;16(4):e1008668. doi: 10.1371/journal.pgen.1008668 (PMC7162527; doi:10.1371/journal.pgen.1008668)
Supplement: S4 Table — (PDF) [file pgen.1008668.s005.pdf]

taxonomic group	tree topologies references
Catarrhine primates	Perelman P, Johnson WE, Roos C, Seuánez HN, Horvath JE, Moreira MA, Kessing B, Pontius J, Roelke M, Rumpler Y, Schneider MP. 2011. A molecular phylogeny of living primates. PLoS genetics. 7(3):e1001342.
Galloanserae	Wright AE, Harrison PW, Zimmer F, Montgomery SH, Pointer MA, Mank JE. 2015. Variation in promiscuity and sexual selection drives avian rate of Faster-Z evolution. Molecular ecology. 24(6):1218-35.
Passeriformes	Barker FK, Barrowclough GF, Groth JG. 2002. A phylogenetic hypothesis for passerine birds: taxonomic and biogeographic implications of an analysis of nuclear DNA sequence data. Proceedings of the Royal Society B: Biological Sciences 269:295–308.
Muroidea	Steppan SJ, Adkins RM, Anderson J, Thorne J. 2004. Phylogeny and Divergence-Date Estimates of Rapid Radiations in Muroid Rodents Based on Multiple Nuclear Genes. Systematic Biology 53:533–553.
Mussels	Distel DL. 2000. Phylogenetic Relationships among Mytilidae (Bivalvia): 18S rRNA Data Suggest Convergence in Mytilid Body Plans. Molecular Phylogenetics and Evolution 15:25–33.
Satyrinae butterflies	Peña C, Wahlberg N, Weingartner E, Kodandaramaiah U, Nylin S, Freitas AV, Brower AV. 2006. Higher level phylogeny of Satyrinae butterflies (Lepidoptera: Nymphalidae) based on DNA sequence data. Molecular phylogenetics and evolution. 40(1):29-49.
Formica ants	J. Romiguier, J. Rolland, C. Morandin, L. Keller. 2018. Phylogenomics of palearctic Formica species suggests a single origin of temporary parasitism and gives insights to the evolutionary pathway toward slave-making behaviour. BMC evolutionary biology 18(1):40.
Earth worms	R. A. King, A. L. Tibble, W. O. C. Symondson. 2008. Opening a can of worms: unprecedented sympatric cryptic diversity within British lumbricid earthworms. *Molecular Ecology* 17, 4684-4698.
Nemertea	Thollesson, Mikael, and Jon L. Norenburg. 2003. Ribbon worm relationships: a phylogeny of the phylum Nemertea. Proceedings of the Royal Society of London B: Biological Sciences 27(1513): 407-415.
Drosophila	Obbard DJ, Maclennan J, Kim KW, Rambaut A, O'grady PM, Jiggins FM. 2012. Estimating divergence dates and substitution rates in the Drosophila phylogeny. Molecular Biology and Evolution. 29(11):3459-73.

S4 Table : Sources of the tree topologies of each taxonomic group used to estimate branch length and map substitutions.
