# Supplementary material for: Is adaptation limited by mutation? A timescale-dependent effect of genetic diversity on the adaptive substitution rate in animals
Source: PLoS Genet. 2020 Apr 6;16(4):e1008668. doi: 10.1371/journal.pgen.1008668 (PMC7162527; doi:10.1371/journal.pgen.1008668)
Supplement: S5 Table — (DOC) [file pgen.1008668.s006.doc]

| **Species** | **Propagule size (cm)** | **source** |
| --- | --- | --- |
| *Formica fusca* | 14 | Forel 1890 |
| *Formica sanguinea* | 10 | Forel 1909 |
| *Formica cunicularia* | 8.5 | Collingwood 1979 |
| *Formica pratensis* | 10.4 | Bolton 1995 |
| *Melanargia galathea* | 0.102 | García-Barros 2000 |
| *Maniola jurtina* | 0.0535 | García-Barros 2000 |
| *Aphantopus hyperantus* | 0.0792 | García-Barros 2000 |
| *Pyronia tithonus* | 0.0628 | García-Barros 2000 |
| *Pyronia bathseba* | 0.0802 | García-Barros 2000 |
| *Mytilus californianus* | 0.01 | Bayne et al. 1983 |
| *Mytilus trossulus* | 0.01 | Bayne et al. 1983 |
| *Mytilus galloprovincialis* | 0.01 | Bayne et al. 1983 |
| *Mytilus edulis* | 0.01 | Bayne et al. 1983 |
| *Allolobophora chlorotica L1* | 0.0238 | Eijsackers 2011 |
| *Allolobophora chlorotica L2* | 0.0238 | Eijsackers 2011 |
| *Allolobophora chlorotica L4* | 0.0238 | Eijsackers 2011 |
| *Aporrecta icterica* | 0.0411 | Eijsackers 2011 |
| *Lumbricus terrestris* | 0.5 | Cloudsley-Thompson and Sankey 1961 |
| *Lineus lacteus* | 0.02 | Bierne, 1983 |
| *Lineus longissimus* | 0.02 | Bierne, 1983 |
| *Lineus sanguineaus* | 0.02 | Bierne, 1983 |
| *Lineus ruber* | 0.5 | Bierne, 1983 |
| *Homo sapiens* | 93.4 | De Magalhaes and Costa 2009 |
| *Pan troglodytes* | 45.12 | De Magalhaes and Costa 2009 |
| *Gorilla gorilla* | 78 | De Magalhaes and Costa 2009 |
| *Papio anubis* | 55.95 | De Magalhaes and Costa 2009 |
| *Pongo abelii* | 42.48 | De Magalhaes and Costa 2009 |
| *Macaca mulatta* | 31.1 | De Magalhaes and Costa 2009 |
| *Anas platyrhynchos* | 55 | Del Hoyo et al. 1992 |
| *Anser cygnoides* | 87 | Del Hoyo et al. 1992 |
| *Meleagris gallopavo* | 90 | Del Hoyo et al. 1992 |
| *Numida meleagris* | 53 | Del Hoyo et al. 1992 |
| *Pavo cristatus* | 95 | Del Hoyo et al. 1992 |
| *Phasianus colchicus* | 57.5 | Del Hoyo et al. 1992 |
| *Parus major* | 13.5 | Del Hoyo et al. 1992 |
| *Ficedula albicollis* | 13 | Del Hoyo et al. 1992 |
| *Corvus sp.* | 50.5 | Del Hoyo et al. 1992 |
| *Geospiza difficilis* | 11.5 | Del Hoyo et al. 1992 |
| *Taniopygia guttata* | 10 | Del Hoyo et al. 1992 |
| *Rattus norvegicus* | 13.717 | De Magalhaes and Costa 2009 |
| *Microtus arvalis* | 7.184 | De Magalhaes and Costa 2009 |
| *Microtus ochrogaster* | 10.6 | De Magalhaes and Costa 2009 |
| *Mus musculus musculus* | 6.06 | De Magalhaes and Costa 2009 |
| *Mus spretus* | 6.37 | Inferred from De Magalhaes and Costa 2009 |
| *Drosophila melanogaster* | 0.0525 | Lott et al. 2007 |
| *Drosophila teissieri* | NA | NA |
| *Drosophila santomea* | NA | NA |
| *Drosophila yakuba* | 0.0475 | Lott et al. 2007 |
| *Drosophila simulans* | 0.05 | Lott et al. 2007 |
| *Drosophila sechellia* | 0.06 | Lott et al. 2007 |

| **Species** | **adult size (cm)** | **source** |
| --- | --- | --- |
| *Formica fusca* | 14 | Forel 1890 |
| *Formica sanguinea* | 10 | Forel 1909 |
| *Formica cunicularia* | 8.5 | Collingwood 1979 |
| *Formica pratensis* | 10.4 | Bolton 1995 |
| *Melanargia galathea* | 2.59 | García-Barros 2000 |
| *Maniola jurtina* | 2.58 | García-Barros 2000 |
| *Aphantopus hyperantus* | 2.12 | García-Barros 2000 |
| *Pyronia tithonus* | 1.89 | García-Barros 2000 |
| *Pyronia bathseba* | 2.04 | García-Barros 2000 |
| *Mytilus californianus* | 7.5 | MArine Life Information Network, 2006 |
| *Mytilus trossulus* | 7.5 | MArine Life Information Network, 2006 |
| *Mytilus galloprovincialis* | 7.5 | MArine Life Information Network, 2006 |
| *Mytilus edulis* | 7.5 | MArine Life Information Network, 2006 |
| *Allolobophora chlorotica L1* | 5.5 | The Trustees of the Natural History Museum,2010 |
| *Allolobophora chlorotica L2* | 5.5 | The Trustees of the Natural History Museum,2010 |
| *Allolobophora chlorotica L4* | 5.5 | The Trustees of the Natural History Museum,2010 |
| *Aporrecta icterica* | 9.5 | Sims and Gerard 1985 |
| *Lumbricus terrestris* | 25 | Cloudsley-Thompson and Sankey 1961 |
| *Lineus lacteus* | 17.5 | Gontcharoff 1951 |
| *Lineus longissimus* | 1000 | Gontcharoff 1951 |
| *Lineus sanguineus* | NA | NA |
| *Lineus ruber* | 5 | Gontcharoff 1951, Bierne 1970 |
| *Homo sapiens* | 163 | Ogden et al. 2004 |
| *Pan troglodytes* | 79.6 | Jones et al. 2009 |
| *Gorilla gorilla* | 137.5 | Wood 1979 |
| *Papio anubis* | 85 | Fleagle 2013 |
| *Pongo abelii* | 83 | Groves 1971 |
| *Macaca mulatta* | 55.5 | Jones et al. 2009 |
| *Anas platyrhynchos* | 55 | Del Hoyo et al. 1992 |
| *Anser cygnoides* | 87 | Del Hoyo et al. 1992 |
| *Meleagris gallopavo* | 90 | Del Hoyo et al. 1992 |
| *Numida meleagris* | 53 | Del Hoyo et al. 1992 |
| *Pavo cristatus* | 95 | Del Hoyo et al. 1992 |
| *Phasianus colchicus* | 57.5 | Del Hoyo et al. 1992 |
| *Parus major* | 13.5 | Del Hoyo et al. 1992 |
| *Ficedula albicollis* | 13 | Del Hoyo et al. 1992 |
| *Corvus sp.* | 50.5 | Del Hoyo et al. 1992 |
| *Geospiza difficilis* | 11.5 | Del Hoyo et al. 1992 |
| *Taniopygia guttata* | 10 | Del Hoyo et al. 1992 |
| *Rattus norvegicus* | 21.5 | Burton and Burton 2002 |
| *Microtus arvalis* | 11.1 | Jones et al. 2009 |
| *Microtus ochrogaster* | 15.2 | Jones et al. 2009 |
| *Mus musculus musculus* | 8 | Berry 1970 |
| *Mus spretus* | 8.6 | Palomo et al. 2009 |
| *Drosophila melanogaster* | 0.85 | Pitnick et al. 2002 |
| *Drosophila teissieri* | NA | NA |
| *Drosophila santomea* | NA | NA |
| *Drosophila yakuba* | NA | NA |
| *Drosophila simulans* | 1.03 | NA |
| *Drosophila sechellia* | NA | NA |

| **Species** | **body mass (g)** | **source** |
| --- | --- | --- |
| *Formica fusca* | NA | NA |
| *Formica sanguinea* | NA | NA |
| *Formica cunicularia* | NA | NA |
| *Formica pratensis* | 0.0119 | Keller & Passera, 1989 |
| *Melanargia galathea* | NA | NA |
| *Maniola jurtina* | 0.05 | Svärd &Wiklund,1989 |
| *Aphantopus hyperantus* | 0.0376 | Svärd &Wiklund,1989 |
| *Pyronia tithonus* | 0,04 | Corbet, 2000 |
| *Pyronia bathseba* | NA | NA |
| *Mytilus californianus* | 37.5 | MArine Life Information Network, 2006 |
| *Mytilus trossulus* | 37.5 | MArine Life Information Network 2006 |
| *Mytilus galloprovincialis* | 37.5 | MArine Life Information Network, 2006 |
| *Mytilus edulis* | 37.5 | MArine Life Information Network, 2006 |
| *Allolobophora chlorotica L1* | 0.3 | Butt 1997 |
| *Allolobophora chlorotica L2* | 0.3 | Butt 1997 |
| *Allolobophora chlorotica L4* | 0.3 | Butt 1997 |
| *Aporrecta icterica* | 0.95 | Bouché 1972 |
| *Lumbricus terrestris* | 7.5 | Quillin, 1999 |
| *Lineus lacteus* | NA | NA |
| *Lineus longissimus* | NA | NA |
| *Lineus sanguineaus* | NA | NA |
| *Lineus ruber* | NA | NA |
| *Homo sapiens* | 62000 | De Magalhaes and Costa 2009 |
| *Pan troglodytes* | 45000 | De Magalhaes and Costa 2009 |
| *Gorilla gorilla* | 93000 | De Magalhaes and Costa 2009 |
| *Papio anubis* | 14700 | De Magalhaes and Costa 2009 |
| *Pongo abelii* | 45000 | De Magalhaes and Costa 2009 |
| *Macaca mulatta* | 8240 | De Magalhaes and Costa 2009 |
| *Anas platyrhynchos* | 1027 | De Magalhaes and Costa 2009 |
| *Anser cygnoides* | 3150 | De Magalhaes and Costa 2009 |
| *Meleagris gallopavo* | 4000 | De Magalhaes and Costa 2009 |
| *Numida meleagris* | 1479 | De Magalhaes and Costa 2009 |
| *Pavo cristatus* | 3375 | De Magalhaes and Costa 2009 |
| *Phasianus colchicus* | 999 | De Magalhaes and Costa 2009 |
| *Parus major* | 17 | Del Hoyo et al. 1992 |
| *Ficedula albicollis* | 12 | Del Hoyo et al. 1992 |
| *Corvus sp.* | 499 | Del Hoyo et al. 1992 |
| *Geospiza difficilis* | 16.15 | Del Hoyo et al. 1992 |
| *Taniopygia guttata* | 10 | Del Hoyo et al. 1992 |
| *Rattus norvegicus* | 320 | De Magalhaes and Costa 2009 |
| *Microtus arvalis* | 27.5 | De Magalhaes and Costa 2009 |
| *Microtus ochrogaster* | 50 | De Magalhaes and Costa 2009 |
| *Mus musculus musculus* | 20.5 | De Magalhaes and Costa 2009 |
| *Mus spretus* | 17 | Palomo et al. 2009 |
| *Drosophila melanogaster* | 0.00115 | Klok et al. 2009 |
| *Drosophila teissieri* | NA | NA |
| *Drosophila santomea* | NA | NA |
| *Drosophila yakuba* | NA | NA |
| *Drosophila simulans* | NA | NA |
| *Drosophila sechellia* | NA | NA |

| **Species** | **Fecundity (number of offspring per year)** | **source** |
| --- | --- | --- |
| *Formica fusca* | NA | NA |
| *Formica sanguinea* | NA | NA |
| *Formica cunicularia* | NA | NA |
| *Formica pratensis* | NA | NA |
| *Melanargia galathea* | NA | NA |
| *Maniola jurtina* | NA | NA |
| *Aphantopus hyperantus* | 140 | Lafranchis et al. 2015 |
| *Pyronia tithonus* | 125 | Lafranchis et al. 2015 |
| *Pyronia bathseba* | NA | NA |
| *Mytilus californianus* | 110000 | MArine Life Information Network, 2006 |
| *Mytilus trossulus* | 110000 | MArine Life Information Network, 2006 |
| *Mytilus galloprovincialis* | 110000 | MArine Life Information Network, 2006 |
| *Mytilus edulis* | 110000 | MArine Life Information Network, 2006 |
| *Allolobophora chlorotica L1* | 0.74 | Edwards & Bohlen 1996 |
| *Allolobophora chlorotica L2* | 0.74 | Edwards & Bohlen 1996 |
| *Allolobophora chlorotica L4* | 0.74 | Edwards & Bohlen 1996 |
| *Aporrecta icterica* | 2.67 | Booth et al. 2000 |
| *Lumbricus terrestris* | NA | NA |
| *Lineus lacteus* | NA | NA |
| *Lineus longissimus* | NA | NA |
| *Lineus sanguineaus* | NA | NA |
| *Lineus ruber* | NA | NA |
| *Homo sapiens* | 0.0008219178 | De Magalhaes and Costa 2009 |
| *Pan troglodytes* | 0.0005479452 | De Magalhaes and Costa 2009 |
| *Gorilla gorilla* | 0.0008219178 | De Magalhaes and Costa 2009 |
| *Papio anubis* | 0.002191781 | De Magalhaes and Costa 2009 |
| *Pongo abelii* | 0.0005479452 | De Magalhaes and Costa 2009 |
| *Macaca mulatta* | 0.002739726 | De Magalhaes and Costa 2009 |
| *Anas platyrhynchos* | 0.02465753 | De Magalhaes and Costa 2009 |
| *Anser cygnoides* | NA | NA |
| *Meleagris gallopavo* | 0.03013699 | De Magalhaes and Costa 2009 |
| *Numida meleagris* | 0.02465753 | De Magalhaes and Costa 2009 |
| *Pavo cristatus* | 0.01369863 | De Magalhaes and Costa 2009 |
| *Phasianus colchicus* | 0.03013699 | De Magalhaes and Costa 2009 |
| *Parus major* | 0.0205 | Tomás et al. 2012 |
| *Ficedula albicollis* | 0.0178 | Gill and Donsker 2017 |
| *Corvus sp.* | 0.01068 | Holyoak 1967 |
| *Geospiza difficilis* | 0.0329 | Grant and Grant 1980 |
| *Taniopygia guttata* | 0.0151 | Olson et al. 2014 |
| *Rattus norvegicus* | 0.1003562 | De Magalhaes and Costa 2009 |
| *Microtus arvalis* | 0.0768 | De Magalhaes and Costa 2009 |
| *Microtus ochrogaster* | 0.04164384 | De Magalhaes and Costa 2009 |
| *Mus musculus musculus* | 0.104 | De Magalhaes and Costa 2009 |
| *Mus spretus* | NA | NA |
| *Drosophila melanogaster* | 6.3 | Hanson et al. 1929 |
| *Drosophila teissieri* | NA | NA |
| *Drosophila santomea* | NA | NA |
| *Drosophila yakuba* | NA | NA |
| *Drosophila simulans* | NA | NA |
| *Drosophila sechellia* | NA | NA |

| **Species** | **Longevity (years)** | **source** |
| --- | --- | --- |
| *Formica fusca* | 20 | Personnal communication |
| *Formica sanguinea* | 20 | Personnal communication |
| *Formica cunicularia* | 20 | Personnal communication |
| *Formica pratensis* | 6 | Personnal communication |
| *Melanargia galathea* | 1 | Lafranchis et al. 2015 |
| *Maniola jurtina* | 1 | Lafranchis et al. 2015 |
| *Aphantopus hyperantus* | 1 | Lafranchis et al. 2015 |
| *Pyronia tithonus* | 1 | Lafranchis et al. 2015 |
| *Pyronia bathseba* | NA | NA |
| *Mytilus californianus* | 25 | Bayne and Bayne 1976 |
| *Mytilus trossulus* | 25 | Bayne and Bayne 1976 |
| *Mytilus galloprovincialis* | 25 | Bayne and Bayne 1976 |
| *Mytilus edulis* | 25 | Bayne and Bayne 1976 |
| *Allolobophora chlorotica L1* | 1.25 | Edwards & Bohlen 1996 |
| *Allolobophora chlorotica L2* | 1.25 | Edwards & Bohlen 1996 |
| *Allolobophora chlorotica L4* | 1.25 | Edwards & Bohlen 1996 |
| *Aporrecta icterica* | NA | NA |
| *Lumbricus terrestris* | NA | NA |
| *Lineus lacteus* | NA | NA |
| *Lineus longissimus* | NA | NA |
| *Lineus sanguineaus* | NA | NA |
| *Lineus ruber* | NA | NA |
| *Homo sapiens* | 123 | De Magalhaes and Costa 2009 |
| *Pan troglodytes* | 59.4 | De Magalhaes and Costa 2009 |
| *Gorilla gorilla* | 60.1 | De Magalhaes and Costa 2009 |
| *Papio anubis* | 37.5 | De Magalhaes and Costa 2009 |
| *Pongo abelii* | 59 | De Magalhaes and Costa 2009 |
| *Macaca mulatta* | 40 | De Magalhaes and Costa 2009 |
| *Anas platyrhynchos* | 29.1 | De Magalhaes and Costa 2009 |
| *Anser cygnoides* | 31 | De Magalhaes and Costa 2009 |
| *Meleagris gallopavo* | 13 | De Magalhaes and Costa 2009 |
| *Numida meleagris* | NA | NA |
| *Pavo cristatus* | 23.2 | De Magalhaes and Costa 2009 |
| *Phasianus colchicus* | 27 | De Magalhaes and Costa 2009 |
| *Parus major* | 15.4 | De Magalhaes and Costa 2009 |
| *Ficedula albicollis* | 9.8 | De Magalhaes and Costa 2009 |
| *Corvus sp.* | 19.2 | De Magalhaes and Costa 2009 |
| *Geospiza difficilis* | 9 | Oschadleus et al. 2016 |
| *Taniopygia guttata* | 12 | De Magalhaes and Costa 2009 |
| *Rattus norvegicus* | 3.8 | De Magalhaes and Costa 2009 |
| *Microtus arvalis* | 4.8 | De Magalhaes and Costa 2009 |
| *Microtus ochrogaster* | 5.3 | De Magalhaes and Costa 2009 |
| *Mus musculus musculus* | 4 | De Magalhaes and Costa 2009 |
| *Mus spretus* | NA | NA |
| *Drosophila melanogaster* | 0.16 | Linford et al. 2013 |
| *Drosophila teissieri* | NA | NA |
| *Drosophila santomea* | NA | NA |
| *Drosophila yakuba* | NA | NA |
| *Drosophila simulans* | NA | NA |
| *Drosophila sechellia* | NA | NA |

**References :**

1. Bayne BL, Salkeld PN, Worrall CM. 1983. Reproductive effort and value in different populations of the marine mussel, Mytilus edulis L. Oecologia 59:18–26.
2. Bayne Brian Leicester, Bayne Brian L. 1976. Marine mussels: their ecology and physiology. Cambridge University Press
3. Berry RJ. 1970. The natural history of the house mouse. Field studies 3:219–262.
4. Bierne J. 1970. Recherches sur la différenciation sexuelle au cours de l’ontogenèse et de la régénération chez le némertien Lineus ruber (Müller).
5. J. Bierne. in Reprod. Biol. Invertebr. 1 Oogenesis Oviposition Oosorption 146–167 (John Wiley & Sons Ltd, 1983).
6. Bolton B. 1995. A new general catalogue of the ants of the world.
7. Booth LH, Heppelthwaite VJ, O’halloran K. 2000. Growth, development and fecundity of the earthworm Aporrectodea caliginosa after exposure to two organophosphates. In: New Zealand Plant Protection Volume 53, 2000. Proceedings of a conference, Commodore Hotel, Christchurch, New Zealand, 8-10 August 2000. New Zealand Plant Protection Society. p. 221–225.
8. Bouché MB. 1972. Lombriciens de France: écologie et systématique.
9. Burton M, Burton R. 2002. International Wildlife Encyclopedia: Index volume. Marshall Cavendish
10. Butt KR. 1997. Reproduction and growth of the earthworm Allolobophora chlorotica(Savingy, 1826) in controlled environments. Pedobiologia 41:369–374.
11. Cloudsley-Thompson JL, Sankey J. 1961. Land invertebrates. A guide to British worms, molluscs and arthropods (excluding insects).
12. Collingwood CA. 1979. The Formicidae (Hymenoptera) of Fennoscandia and Denmark. Scandinavian Science Press.
13. Corbet SA. 2000. Butterfly nectaring flowers: butterfly morphology and flower form. *Entomologia Experimentalis et Applicata*, *96*:289-298.
14. De Magalhaes JP, Costa J. 2009. A database of vertebrate longevity records and their relation to other life‐history traits. Journal of evolutionary biology 22:1770–1774.
15. Del Hoyo J, Elliot A, Sargatal J. 1992. Handbook of the Birds of the World. Barcelona: Lynx Editions.”. Jutglar, Francesc.
16. Edwards CA, Bohlen P J. 1996. Biology and Ecology of Earthworms. London: Chapman and Hall.
17. Eijsackers H. 2011. Earthworms as colonizers of natural and cultivated soil environments. Applied Soil Ecology 50:1–13.
18. Fleagle JG. 2013. Primate adaptation and evolution. Academic Press.
19. Forel A. 1890. Fourmis de Tunisie et de l’Algérie orientale. Annales.
20. Forel A. 1909. Fourmis d’Espagne. Récoltées par MO Vogt et Mme Cécile Vogt, docteurs en médecine. Annales.
21. García-Barros E. 2000. Egg size in butterflies (Lepidoptera: Papilionoidea and Hesperiidae): a summary of data. Journal of Research on the Lepidoptera 35:90–136.
22. Gill F, Donsker D. 2017. IOC World Bird List (v 7.2), 10.14344/IOC. ML
23. Gontcharoff M. 1951. Biologie de la régénération et de la reproduction chez quelques Lineidae de France. Annales des Sciences Naturelles, Zoologie, Serie 11 13:149–235.
24. Grant PR, Grant BR. 1980. The breeding and feeding characteristics of Darwin’s finches on Isla Genovesa, Galapagos. Ecological Monographs 50:381–410.
25. Groves CP. 1971. Pongo pygmaeus. Mammalian species:1–6.
26. Hanson FB, Ferris FR.1929. A quantitative study of fecundity in Drosophila melanogaster. Journal of Experimental Zoology. 543:485-506.
27. Holyoak D. 1967. Breeding biology of the Corvidae. Bird Study 14:153–168.
28. Jones KE, Bielby J, Cardillo M, Fritz SA, O’Dell J, Orme CDL, Safi K, Sechrest W, Boakes EH, Carbone C. 2009. PanTHERIA: a species‐level database of life history, ecology, and geography of extant and recently extinct mammals. Ecology 90:2648–2648.
29. Keller, L. and Passera, L., 1989. Size and fat content of gynes in relation to the mode of colony founding in ants (Hymenoptera; Formicidae). *Oecologia*, *80*2 :236-240.
30. Klok CJ, Hubb AJ, Harrison JF. 2009. Single and multigenerational responses of body mass to atmospheric oxygen concentrations in Drosophila melanogaster: evidence for roles of plasticity and evolution. Journal of evolutionary biology. 12:2496-504.
31. Lafranchis, T., Jutzeler, D., Guillosson, J.Y., Kan, P. and Kan, B. 2015. La vie des papillons: écologie, biologie et comportement des Rhopalocères de France. Diatheo.
32. Linford NJ, Bilgir C, Ro J, Pletcher SD. 2013. Measurement of lifespan in Drosophila melanogaster. Journal of visualized experiments 71.
33. Lott SE, Kreitman M, Palsson A, Alekseeva E, Ludwig MZ. 2007. Canalization of segmentation and its evolution in *Drosophila*. Proceedings of the National Academy of Sciences. 10426:10926-31.
34. MArine Life Information Network. MarLIN BIOTIC (Biological Traits Information Catalogue).(2006). at <[www.marlin.ac.uk/biotic/](http://www.marlin.ac.uk/biotic/)>
35. Ogden CL, Fryar CD, Carroll MD, Flegal KM. 2004. Mean body weight, height, and body mass index: United States 1960-2002. Department of Health and Human Services, Centers for Disease Control and Prevention, National Center for Health Statistics Washington, DC
36. Olson CR, Wirthlin M, Lovell PV, Mello CV. 2014. Proper care, husbandry, and breeding guidelines for the zebra finch, Taeniopygia guttata. Cold Spring Harbor Protocols 2014:pdb. Prot084780.
37. Oschadleus HD, Schultz B and Schultz SJ. 2016. Longevity of the Helmeted Guineafowl Numida meleagris. *Biodiversity Observations*,1-3.
38. Palomo LJ, Justo ER, Vargas JM. 2009. Mus spretus (Rodentia: muridae). Mammalian species:1–10.
39. Pitnick S, García–González F. 2002. Harm to females increases with male body size in Drosophila melanogaster. Proceedings of the Royal Society of London. Series B: Biological Sciences. 269(1502):1821-8.
40. Quillin KJ. 1999. Kinematic scaling of locomotion by hydrostatic animals: ontogeny of peristaltic crawling by the earthworm Lumbricus terrestris. *Journal of Experimental Biology*, *202 :*661-674.
41. Seifert B. 2002. A taxonomic revision of the Formica cinerea group (Hymenoptera: Formicidae). Abhandlungen und Berichte des Naturkundemuseums Görlitz 74:245–272.
42. Sims RW, Gerard BM. 1985. Earthworms: keys and notes for the identification and study of the species. Brill Archive
43. Svärd L, and C Wiklund. 1989. Mass and production rate of ejaculates in relation to monandry/polyandry in butterflies. *Behavioral Ecology and Sociobiology* 24.6: 395-402.
44. The Trustees of the Natural History Museum. Available from: <http://www.nhm.ac.uk/discover.html>
45. Tomás G, Barba E, Merino S, Martínez J. 2012. Clutch size and egg volume in great tits (Parus major) increase under low intensity electromagnetic fields: a long-term field study. Environmental research 118:40–46.
46. Wood BA. 1979. Relationship between body size and long bone lengths in Pan and Gorilla. American journal of physical anthropology 50:23–25.

**S5 Table : Values and sources of the life history traits used in this study.**
