# Supplementary figures and images for: Is adaptation limited by mutation? A timescale-dependent effect of genetic diversity on the adaptive substitution rate in animals
Source: PLoS Genet. 2020 Apr 6;16(4):e1008668. doi: 10.1371/journal.pgen.1008668 (PMC7162527; doi:10.1371/journal.pgen.1008668)

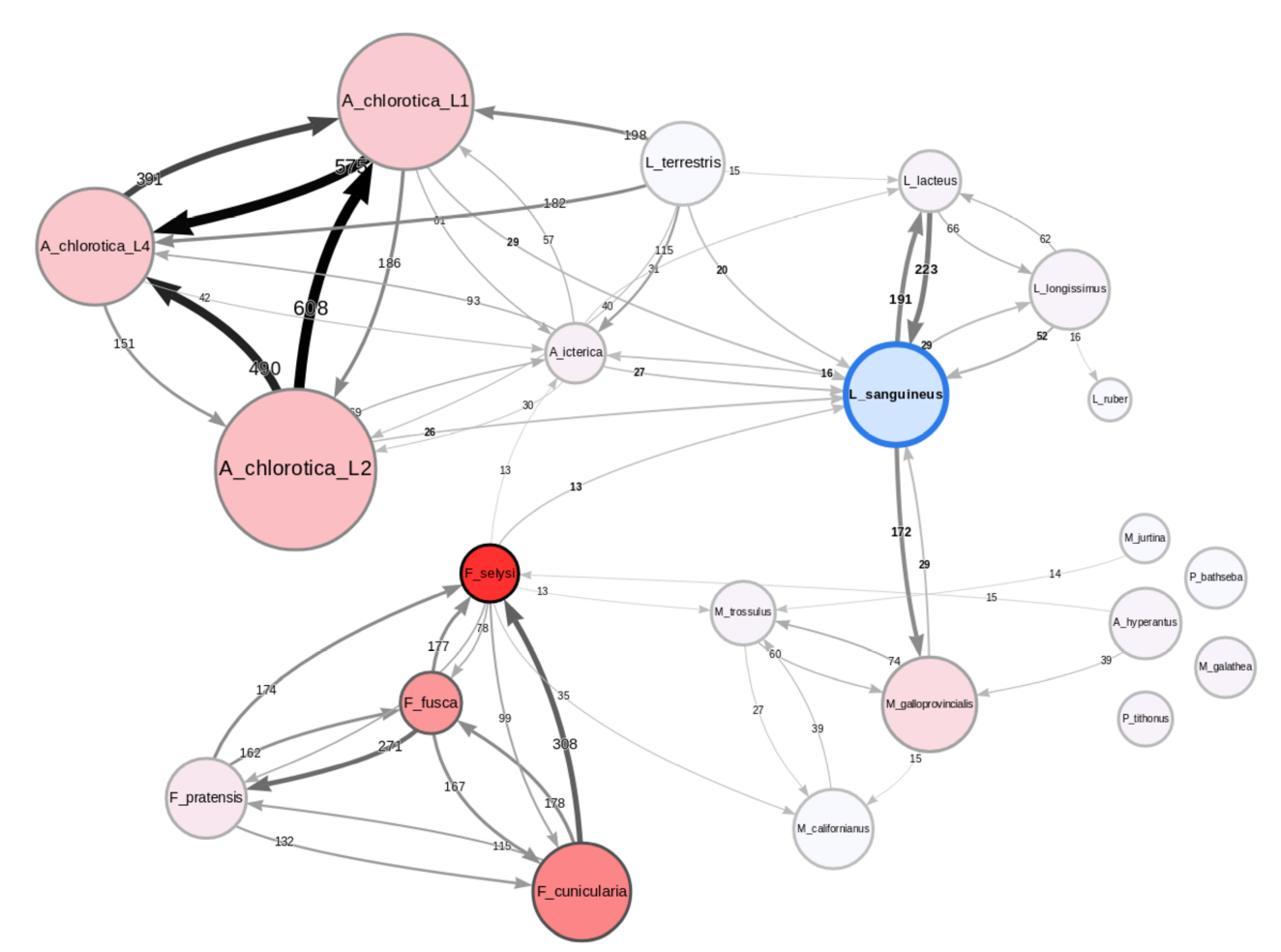

Supplement: S1 Fig — Circles represent the assemblies, and arrows and their corresponding numbers represent the number of cross contaminants. Most cross contamination events occur between closely-related species and are therefore likely false positive cases. (TIF) [file pgen.1008668.s008.tif]

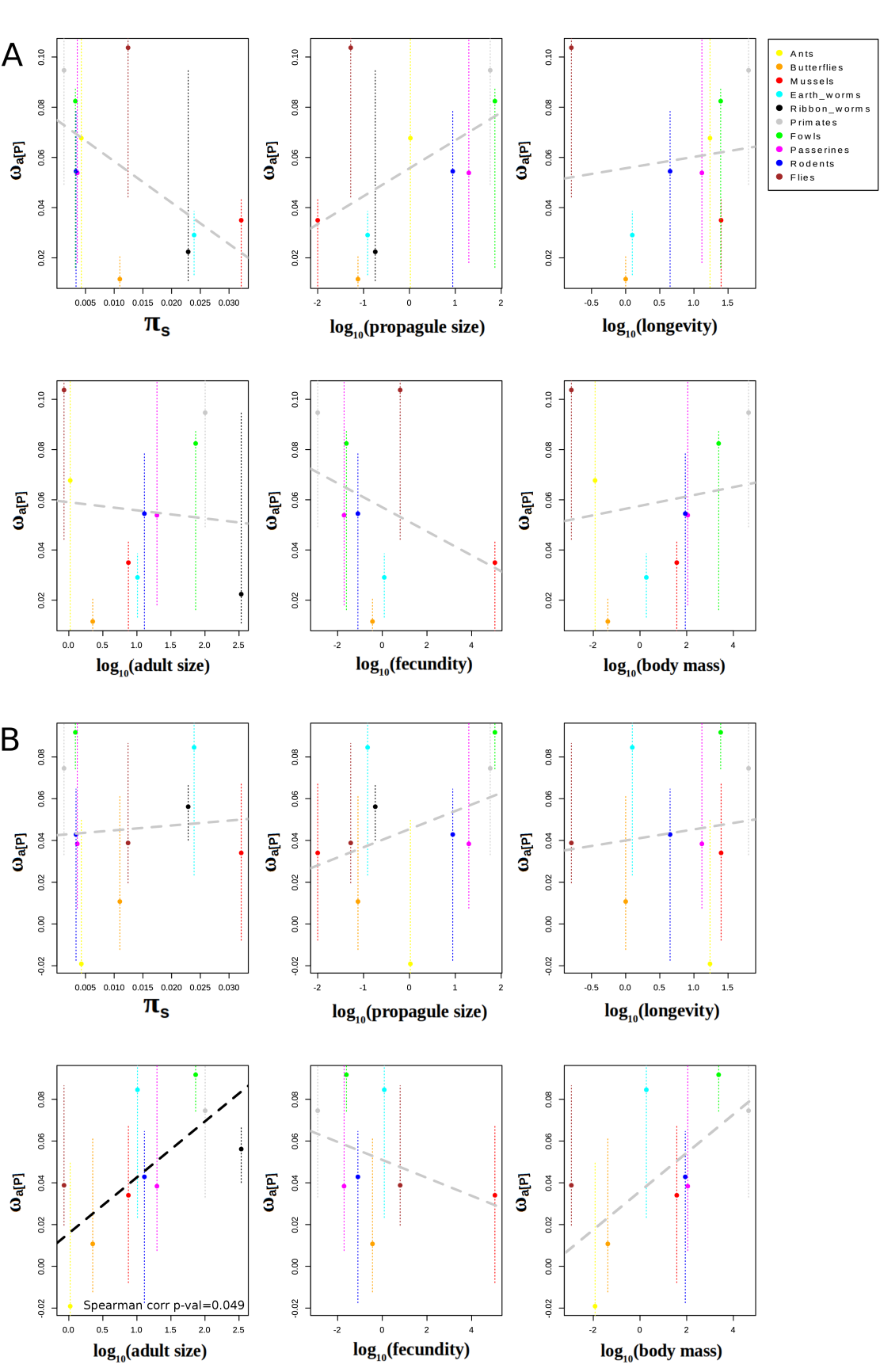

Supplement: S2 Fig — ωa[P] is estimated using all mutations and substitutions (A) or using only GC-conservative mutations and substitutions (B). Group level πs and life history traits are estimated by averaging species level estimates across closely related species. Black dotted lines represent significant regressions across taxonomic groups and grey dotted lines non-significant ones. (TIF) [file pgen.1008668.s009.tif]

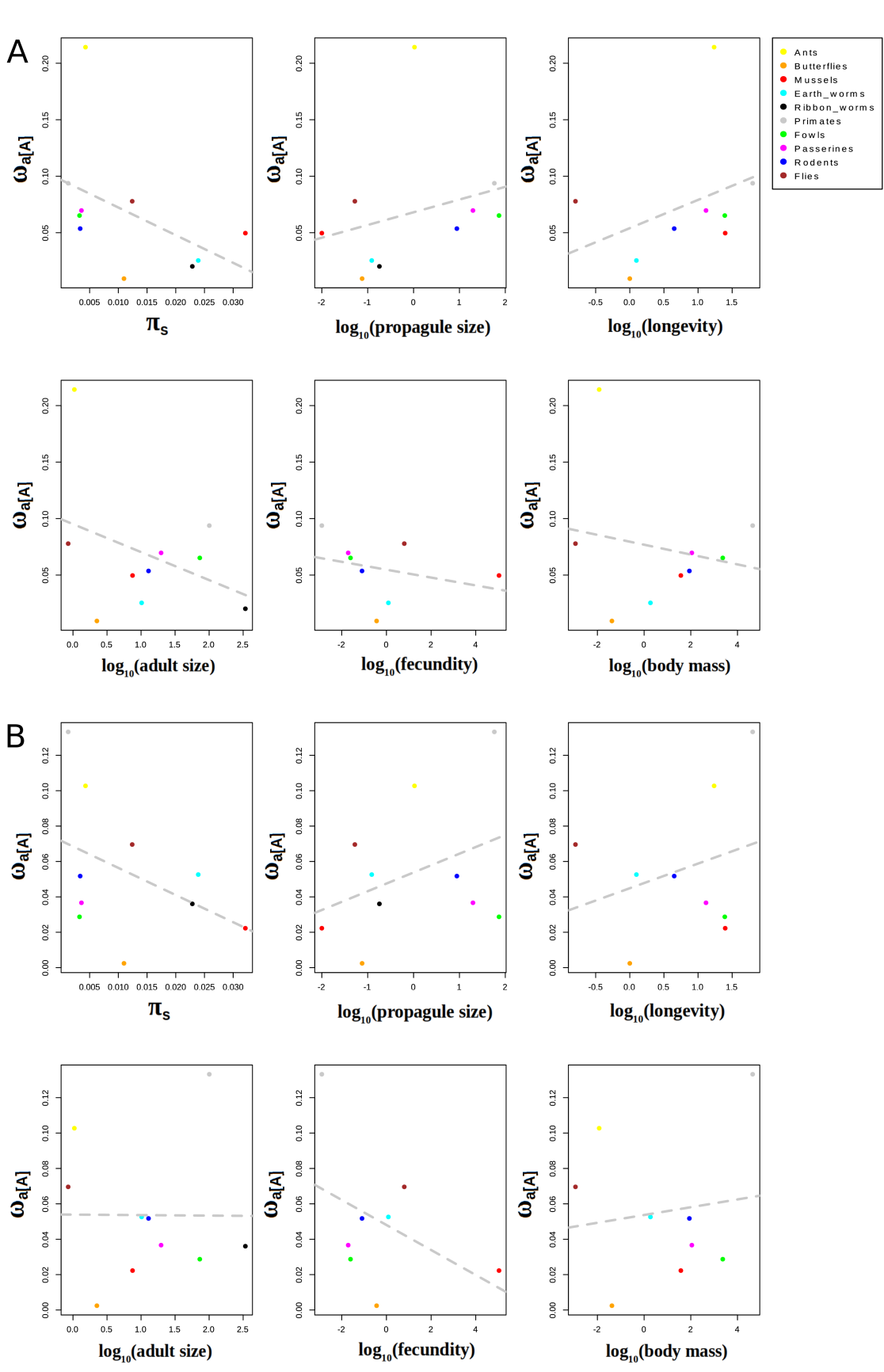

Supplement: S3 Fig — ωa[A] is estimated using all mutations and substitutions (A) or using only GC-conservative mutations and substitutions (B). Group level πs and life history traits are estimated by averaging species level estimates across closely related species. Black dotted lines represent significant regressions across taxonomic groups and grey dotted lines non-significant ones. (TIF) [file pgen.1008668.s010.tif]

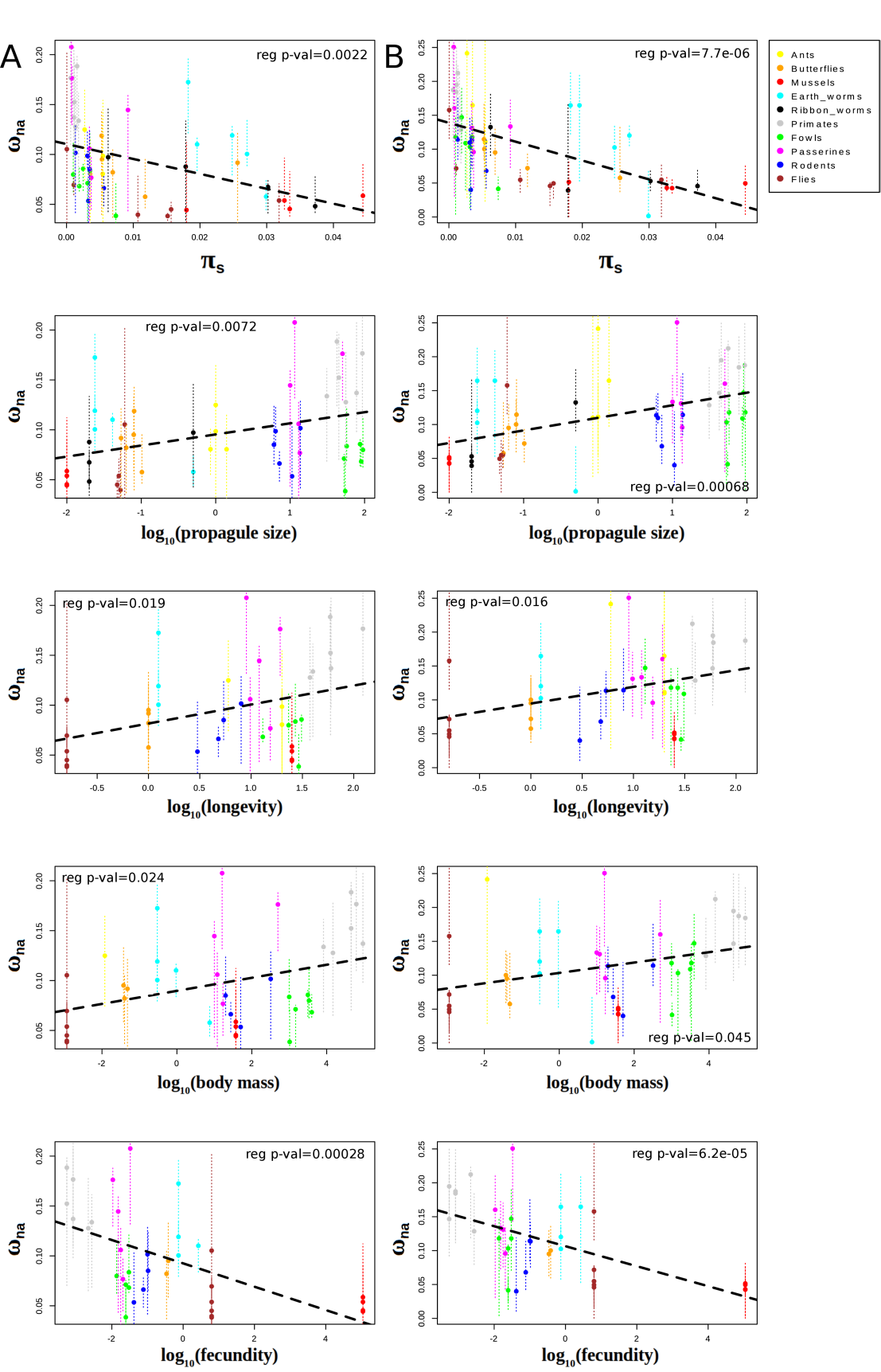

Supplement: S4 Fig — ωna is estimated using all mutations and substitutions (A) or using only GC-conservative mutations and substitutions (B). Black dotted lines represent significant regressions across taxonomic groups and grey dotted lines non-significant ones. (TIF) [file pgen.1008668.s011.tif]

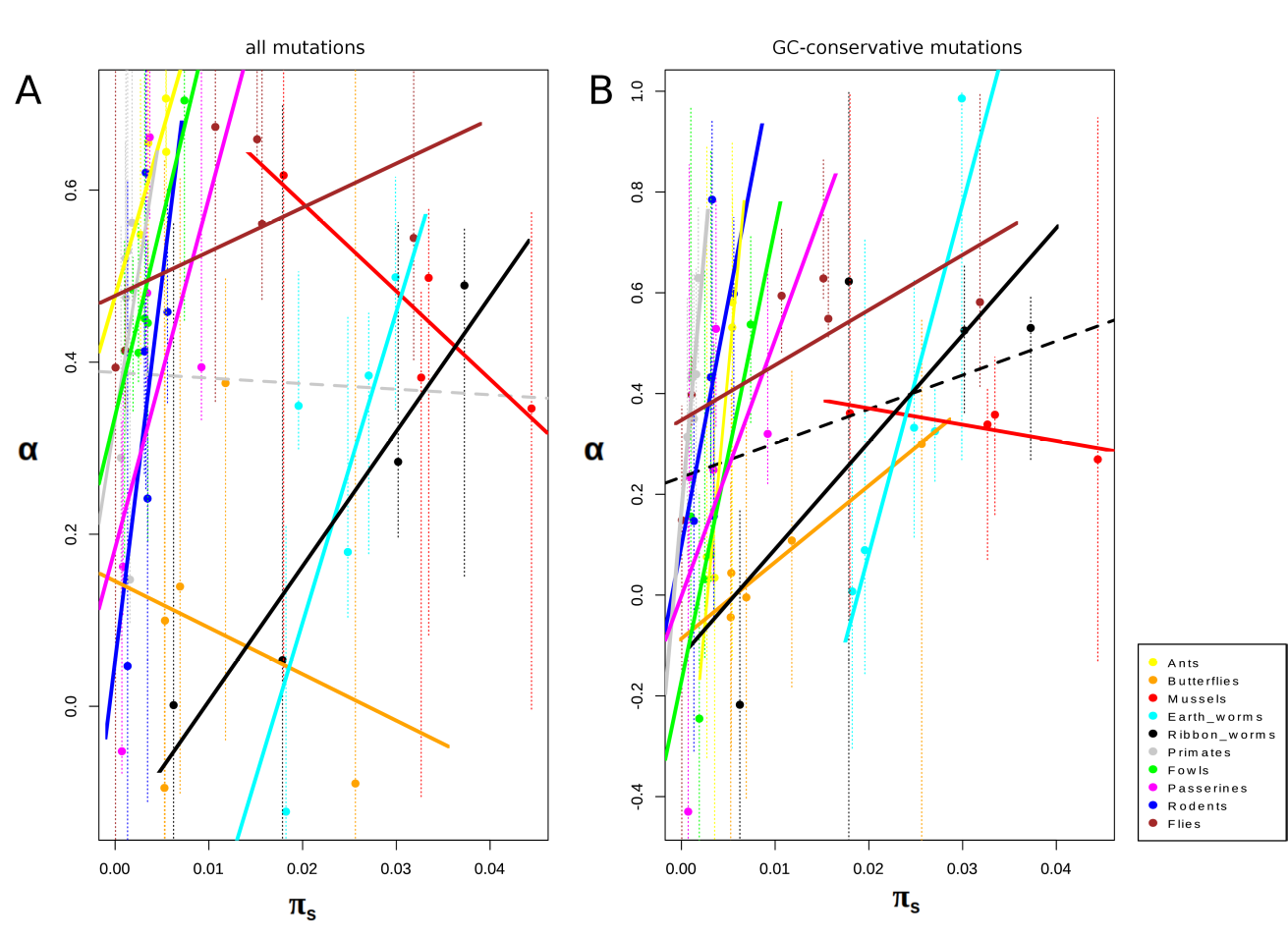

Supplement: S5 Fig — α is estimated using all mutations and substitutions (A) or using only GC-conservative mutations and substitutions (B). The dotted line represents the regression across all species, and full lines represent the regression within each taxonomic groups. Black dotted lines represent significant regressions across taxonomic groups and grey dotted lines non-significant ones. (TIF) [file pgen.1008668.s012.tif]

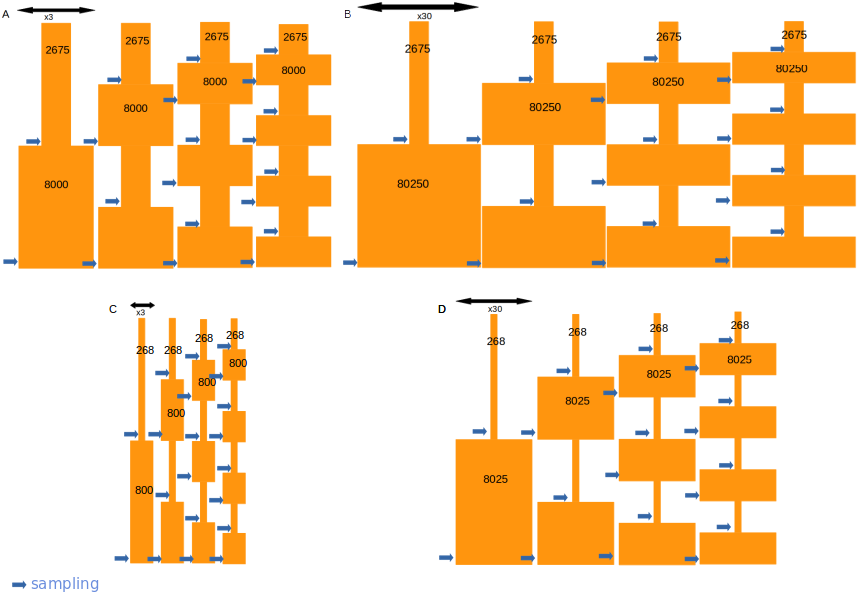

Supplement: S6 Fig — A: three fold ratio between low and high population size and high long-term population size. B: thirty fold ratio between low and high population size and high long-term population size. C: three fold ratio between low and high population size and low long-term population size. D: thirty fold ratio between low and high population size and low long-term population size. (TIF) [file pgen.1008668.s013.tif]

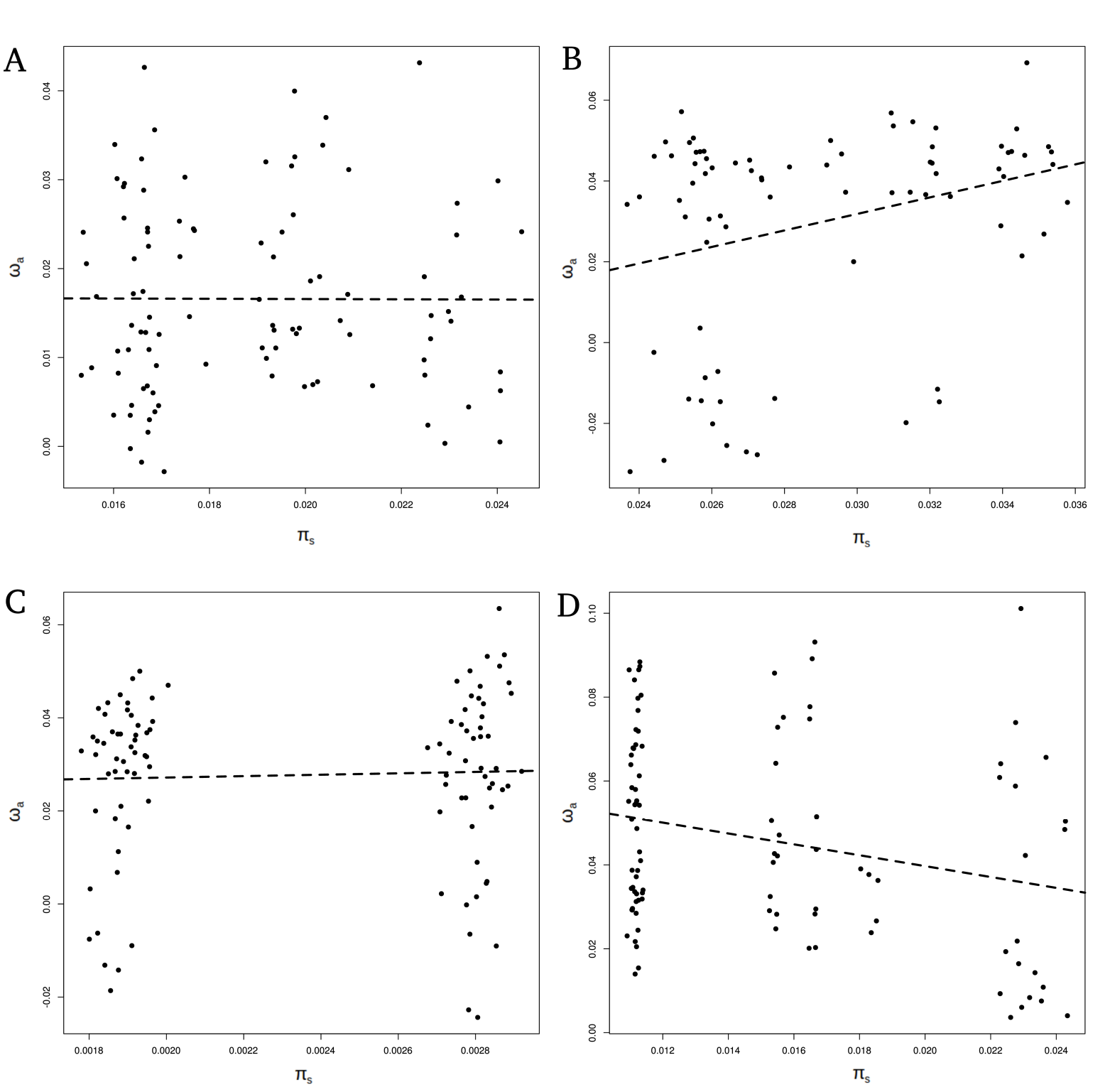

Supplement: S7 Fig — A: three fold ratio between low and high population size and high long-term population size (scenario A in S1 Fig) B: thirty fold ratio between low and high population size and high long-term population size (scenario B in S1 Fig) C: three fold ratio between low and high population size and low long-term population size (scenario C in S1 Fig) D: thirty fold ratio between low and high population size and low long-term population size (scenario D in S1 Fig) (TIF) [file pgen.1008668.s014.tif]
