# Supplementary material for: Is adaptation limited by mutation? A timescale-dependent effect of genetic diversity on the adaptive substitution rate in animals
Source: PLoS Genet. 2020 Apr 6;16(4):e1008668. doi: 10.1371/journal.pgen.1008668 (PMC7162527; doi:10.1371/journal.pgen.1008668)
Supplement: Attachment — Submitted filename: response_to_reviwers.pdf [file pgen.1008668.s015.pdf]

Response to the reviewers for the revision of the article untitled "Is adaptation limited by mutation? A timescale-dependent effect of genetic diversity on the adaptive substitution rate in animals", by Rousselle M, Simion P, Tilak MK, Figuet E, Nabholz B, Galtier N.

Response to David Castellano's comments (reviewer 2):

(1) Folded or unfolded SFS? In section 5 and 6 from the Materials and Methods it is not clear when the folded and the unfolded SFS are used. For the GammaExpo and ScaleBeta models I assume that the unfolded SFS is used, but for the Gamma model both the unfolded and folded can be used. I think that more details about how the data is fitted into these models must be added as this is a critical part of this work. It would be nice to see the likelihood of each model, the value of the inferred parameters and its AIC weight. Maybe as a supplementary table?

Thank you for this suggestion. We added a supplementary table where we report species estimates of life history traits, dN/dS, π_s, Tajima's D and F_{is}, as well as α, ω_a and ω_{na} for each model and model averaged via AIC weights (Table S6).
We used folded SFS in all analyses, and we make this clearer in the manuscript (line 728-730).

(2) Level of uncertainty in the measures. Why is the data not bootstrapped? Bootstrap replicates will give a measure of uncertainty in DFE and omega_a estimates which will be very useful when making regressions and computing p-values.

Right. We added 95% confidence intervals obtained by bootstrapping SNPs (see all figures). We cannot report bootstraps confidence intervals for the averaging method used to obtained one of the two estimates of group level ω_a, which explains the absence of confidence intervals in some panels of Figure 1 and in Figure S3.

(3) Variation in gene density (or recombination rate between selected mutations) vs Fisher's Geometrical Model (FGM). The authors interpret the negative correlation between omega_a and neutral diversity across distantly related groups of species in terms of FGM. So in terms of differences in the DFE between large and small long-term Ne species. I do not disagree with them, FGM is a valid interpretation. However, one could also assume the same DFE across species (which personally I find very unrealistic) and that the variation in omega_a is entirely driven by variation in the intensity of Hill-Robertson interference (HRi). If large Ne species tend to have more compact genomes (high gene density, or less recombination between selected mutations, this seems to be true at least for the comparison Drosophila-primates) they might be under stronger HRi than small Ne species. Second, if most adaptation occurs through small steps (weakly beneficial mutations), then large Ne species will lose more weakly beneficial mutations than small populations. HRi and FGM are not incompatible, I believe it is likely that both are operating and contributing to explain the negative correlation between omega_a and diversity. I think that HRi could be mentioned when discussing point 4 of the discussion.

Thanks for this comment. We added a paragraph related to HRi in the section 4 of the

discussion, where we discuss potential factors that can explain the absence of positive large-scale relationship between the rate of adaptive evolution and genetic diversity (lines 490 to 494).

Minor comments:

(1) Figure 1B and D, Figure 2B and Figure 3B. GC-conservative mutations are less common than non-GC-conservative mutations, why is this not reflected in the x-axis of those Figures? Is the regression done with all mutations or only with GC-conservative mutations? Does this affect the results?

In the result shown, π_s is always estimated using all mutations, hence the fact that there is no difference in the X-axis between plots with all *vs*. only GC-conservative mutations.
We do not necessarily expect that π_s estimated using GC-conservative is smaller than π_s estimated using all mutation, because GC-conservative synonymous sites are also less common than all synonymous sites, which is taken into account in the computation of π_s.
However, it is true that this may influence the results, in particular if gBGC influences the estimation of π_s.
We first checked that $_s$ estimated using all mutation *vs*. π_s estimated using GC-conservative mutations are correlated (r^2=0.55, p-value=4.8e-10).
Additionally, we reproduced some of the analyses using GC-conservative π_s, and we can see that the results are quantitatively unchanged.

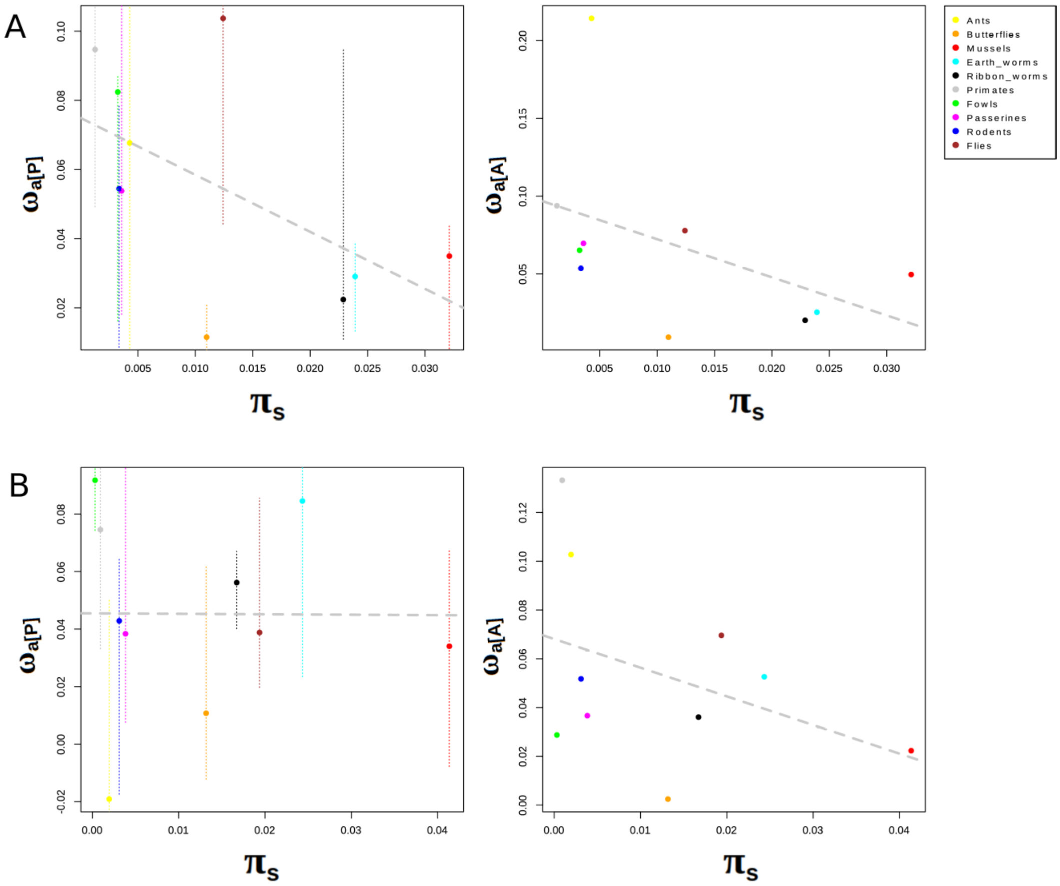

Figure R1: Relationship between group-level ω_a and group-level π_s.

A: ω_a was estimated by pooling SFS across species within a group ($\omega_{a[P]}$) (left) or via the averaging of ω_{na} across species within a group ($\omega_{a[A]}$) (right) using all mutations.

B: ω_a was estimated by pooling SFS across species within a group ($\omega_{a[P]}$) (left) or via the averaging of ω_{na} across species within a group ($\omega_{a[A]}$) (right) using only GC-conservative mutations. π_s was estimated using only GC-conservative mutations.

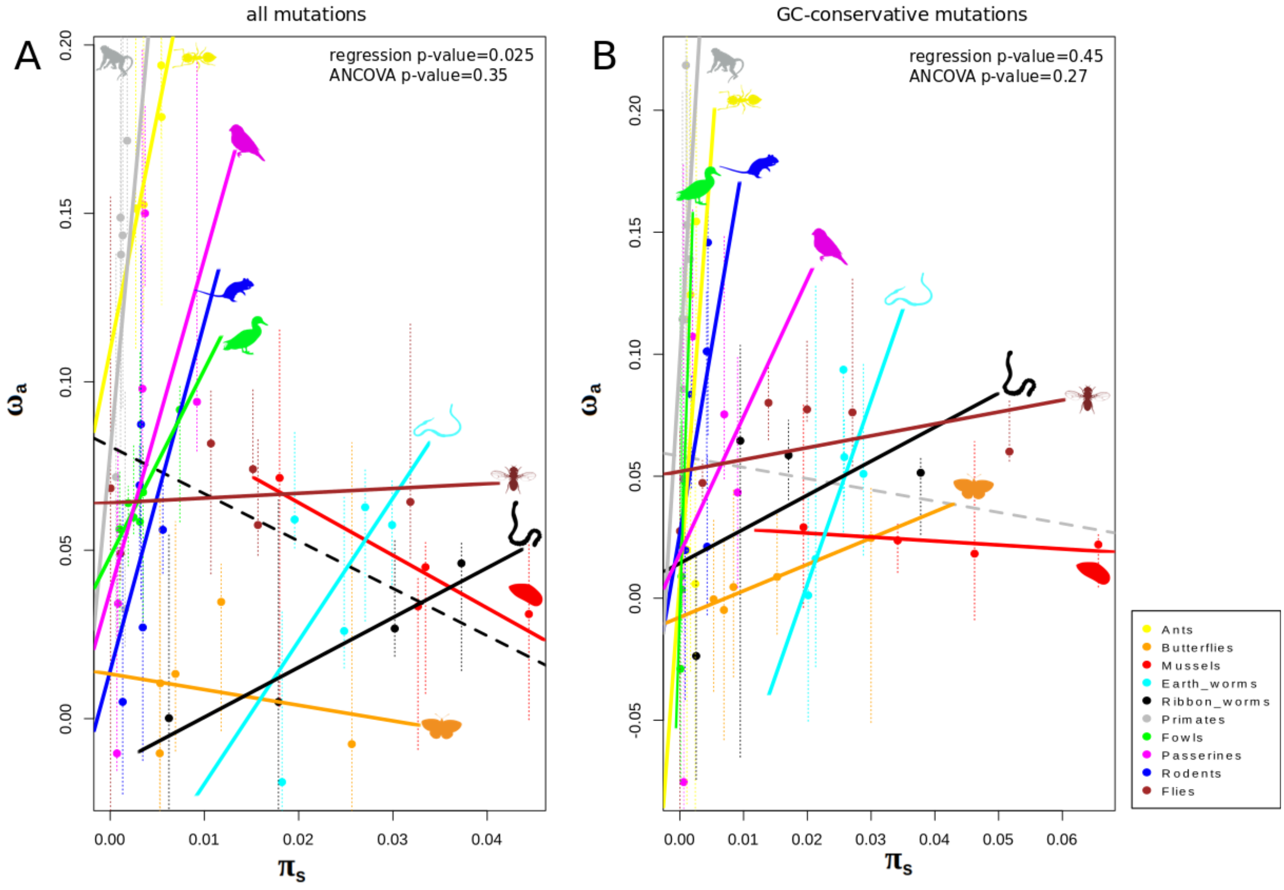

Figure R3: Relationship between species-level ω_a and π_s.

A: ω_a is estimated using all mutations.

B: ω_a and π_s are estimated using only GC-conservative mutations.

Black dotted lines represent significant regressions across taxonomic groups and grey dotted lines non-significant ones.

(2) Line 262. It would be great to briefly explain the expected relation between long-term Ne and the life history traits. It might not be straightforward for everyone to guess the expected relationships.

We added a short paragraph at the beginning of the result section related to life history traits explaining the link between genetic diversity and life-history strategies (lines 263 to 268).

(3) Line 269. Regression equations and p-values are reported inconsistently. Sometimes only the r^2 is reported, sometimes only the p-values, sometimes only the slope (see line 418). I suggest to make a table with all the correlations commented on the text to back up also the main figures. Then the authors can directly refer the reader to the table.

Thanks for this comment. We now consistently report both the r^2 and the p-values when performing regression (per-species analysis), or the correlation coefficient and p-value when performing Spearman correlation test (per-group analysis) (those changes are indicated in yellow in the "track changes" version of the manuscript). Instead of a table, we also now

indicate the p-values when significant in all figures. When regressions are not significant, the regression line in indicated in a grey dotted line.

(4) Line 308. Is this correlation coefficient referring to all mutations or GC-conservative mutations? Note also that there is a contradiction between this sentence and the legend text at Figure 3. Please check.

This is referring to GC-conservative mutations. We made this clearer (line 308 to 311).

(5) Figure 3B. It looks like one mussel species has disappeared, the red line is shorter. Please check.

There is no species missing (there are four mussel species in our dataset), but the mussel regression line was not appropriate in Figure 3A, hence the comment. We modified the figure accordingly.

(6) Line 323. Figure S4 is not equivalent to Figure 3 but with omega_na. Figure S4 is equivalent to Figure 2 instead.

Indeed, we do not show an equivalent to Figure 3 with ω_{na}, but only the global relationship between ω_{na} and π_s in figure S4 A and B. The mention to Figure S4 was misplaced, and we corrected it.

(7) Line 343. F_is statistic. It would be great to explain what this statistic is and a reference to find it.

Right. We now indicate more clearly what F_{is} measures (lines 352 to 356) and we report the estimates in the newly added supplementary Table S6.

(8) Line 358. It should be figure 3 instead of figure 2. I believe that the same applies for lines 370 and 406. Please check.

Thanks for spotting this mistake. We corrected it.

(9) Lines 408-410. Castellano et al. 2019 compared the DFE across closely related species (great apes) finding that the deleterious DFE is quite stable across great apes. This work is in agreement with the assumptions of the current work where the DFE is expected to be similar between closely related species but different between distantly related species.

Thanks for this useful addition. We modified the section accordingly (lines 421 to 424).

(10) Line 710-712. Something weird is going on with the AIC weighting (probably during the pdf conversion).

Thanks for spotting this.

Response to Adam Etre-Walker's comments (reviewer 3):

1. They start by reasoning that species with larger populations should have higher rates of adaptive evolution because they generate more mutations per generation, they are more genetically diverse and selection is more efficient. However, I think one has to be cautious because there are a number of hidden assumptions behind these statements. First, larger populations do indeed generate more mutations per generation, however this rate at which adaptive mutations are generated may not be well captured by an estimate of the neutral population mutation rate. This is because the effective population size as it pertains to neutral variation and the population size generating adaptive mutations might be very different, as Petrov and colleagues have argued. So assuming that the neutral population mutation rate is correlated to the adaptive population mutation rate may not be justified. Furthermore, there is some evidence that the effective population size and mutation are negatively correlated; hence species with high diversity might not be those with high Ne. Second, species with high neutral population mutation rates are more diverse for neutral genetic variation, but this does not mean they are more diverse for genetic variation that selection might act upon; this is because for deleterious genetic variation in which Nes1 the equilibrium frequency does not depend upon the effective population size. Finally, whilst we often assume that the level of neutral genetic diversity is a measure of the effective population size, this need not be the case. Some discussion of these points might be warranted.

Thanks for these comments, with which we fully agree. We have added a paragraph discussing the limitations of using θ as a proxy for the beneficial mutation supply, with appropriate references (lines 455 to 467). Although all these concerns are valid, we note that the relationship between theta and the adaptive rate has been the focus of a number of studies before this one, including several by the reviewer himself. We believe that there is an interest in understanding this complex relationship despite the important caveats mentioned here.

2. As the authors are aware, a challenge in using the MK approach to estimating the rate of adaptive evolution is population size change; if the current Ne, which applies to the polymorphism data, is larger than the ancestral Ne, which applies to the divergence data, then the rate of adaptive evolution is overestimated, and vice versa. The positive correlation between the rate of adaptive evolution and theta could therefore be due to the simple fact that species which are expanding will on average tend to have higher theta than those that are contracting, and will have an estimate of adaptive evolution which is biased upwards. The authors run some simulations to investigate whether this is the case, but I didn't find their simulations particularly convincing; what they need to simulate is a range of demographies in which some

species are expanding and others are contracting. Across this range of demographies do they observe a correlation between the rate of adaptive evolution and theta. The results of such a simulation will depend upon how strong the population size increases and decreases are, versus the variance in the ancestral theta. However, the fact that the slope of the relationship between omega and theta gets weaker as the mean theta increases, suggests to me this model is not correct.

Thanks for this comment, which we think mainly results from a lack of clarity of the first version of our manuscript. Our simulations actually include instances of contracting and expanding populations. This is because we sample at different time points along the process of fluctuating N_e, as now illustrated in the revised figure S6. So we do sample a wide range of recent demographies, which is why, we believe, we indeed detect a (weak) correlation between ω_a and theta when fluctuations are pronounced. The merits we see of our simulation scheme is that it also provides us with a plausible quantification of the effect of varying N_e on dN/dS and its variance, as discussed by the reviewer.

3. The authors find that within each group there is a positive correlation. Between the rate of adaptive evolution and the level of neutral diversity, particularly in species with low diversity. However, between groups this correlation becomes negative. They interpret this in terms of the time-scale; in doing so they seem to be implying that the effect within groups is a consequence of non-equilibrium dynamics, and changes in Ne or neutral diversity. If this is the case then the pattern within a group could also be an artfecatual consequence of changing Ne.

In summary, I think the central observations are very interesting, but I remain unclear whether they have presented a plausible explanation for the observations.

We acknowledge this assessment, also shared by Reviewer 1. In the revised version we make our best to focus on the novel results, and be more prudent as far as interpretation is concerned (e.g. lines 465 to 467).

Response to reviewer 1's comments:

I found the manuscript extremely confusing in its design and the conclusions. First, of all the estimates of diversity are not the same as the population size.

Here we used the synonymous genetic diversity as an estimate of the population mutation rate $\theta=4N_e\mu$, which is used as a proxy for the mutation supply. Our working hypothesis is that if the mutation supply is limiting than the adaptive rate should respond to θ, as suggested by basic/simplistic theoretical arguments (lines 78-80).

We added a paragraph on the merits and limitations of θ as a proxy for the mutation supply (lines 455-467).

Given that higher rate of adaptation can reduce levels of diversity, it seems strange to treat diversity as N and treat wa as an independent variable.

We acknowledge that a higher rate of adaptation can reduce levels of diversity due to linkage, but as we report on the contrary in almost all groups a positive relationship between ω_a and θ (cf. Section 4 of the results: the ANCOVA analysis reveals a significant positive relationship between $\omega_{a[GC\text{-}conservative]}$ and π_s), we can conclude that there must be another mechanism that compensates the effect of linked adaptive mutations on neutral genetic diversity, which we thought interesting to investigate as the central topic of this study.

This is discussed in the new paragraph we wrote (lines 455-467), which also calls for caution with respect to the interpretation of our results.

Relatedly, whatever the relationship one finds it will not tell us whether or not adaptation is limited by mutation. The title is misleading.

We identify limiting mutation supply as a natural explanation to the existence of a positive relationship between theta and the adaptive rate. We would be happy to consider any alternative hypothesis the reviewer would have in mind. We did not modify the manuscript based on this comment.

Second, the relationships found are extremely noisy and strongly dependent on particulars of the analysis and no clarity emerged as I looked through the figures. Figure 1 showed either positive or negative slopes depending on which mutations are used and the slopes are barely distinguishable from zero.

Indeed, the negative large-scale relationship is a weak, negative relationship, as we acknowledge several times in the manuscript. We now alleviated even more this message in the manuscript. However, the absence of a positive relationship is a very interesting result *per se*, we suggest, as it reveals a difference in behavior between small and large taxonomic scale.

In addition some values of wa are below zero which makes me disbelief the whole approach.

These values are explained by the relative high sampling variance associated with MK estimates of the adaptive rate. We now report confidence intervals obtained by bootstrapping the data.

Fig. 2 looks at life history traits with stronger signals. These history traits might actually be reasonable proxies of the population size but the authors do not focus on these.

Here, life history traits are used as alternative explanatory variables of the adaptive substitution rate than the mutation supply.
Part of our discussion on the large taxonomic scale effect (i.e. part 4 of the discussion) is based on the observation that the adaptive substitution rate is negatively correlated with life history traits presumably linked to long-term population size and the generation time.

Finally, Fig. 3 presents us with a L shape data. Some taxa (like primates) have very variable wa but no variation in diversity and others have no variation in the rate of adaptation and much variation in diversity. Basically, it is a mess and no clarity emerges.

This analysis is also valid, as mentioned, when using $\log_{10}$ transformed π_s, alleviating the differences in variation of π_s within groups.
The clarity that emerges here is that the per-species slope of the regression between ω_a and θ are negatively correlated with θ. This is consistent with the hypothesis that the limitation of adaptation by the supply of adaptive mutations is effective and strong in small-θ groups (e.g. primates, rodents, ants), but not in high-θ groups of animals (e.g. flies, mussels, butterflies).

Then the Discussion reefers to Fig. 2 and makes claims that are not in Fig. 2 ("At a recent 357 evolutionary scale (i.e., neutral divergence <0.2 subst./site), we found a significant positive 358 correlation between ωa and πs (Figure 2).")

This is a typo, thanks for spotting it.

Even while I do not understand where the findings really are - my reading is that the authors found basically no pattern, I also don't understand how these results are consistent with Fisher's geometric model. Under equilibrium, it is not obvious what the MK results would be as all adaptive and deleterious mutations will happen at the same rate (Sella and Hirsh result) and should lead to nearly neutral evolution.

We agree. At equilibrium under FGM, the expected adaptive rate *sensu* MK is essentially zero. The suggestion that proteins are further away from their optimum in small-N_e than in large-N_e species, which would tend to increase ω_a in the former, has been made by Huber et al (2017), as we now explicitly state. This, we think, is an interesting hypothesis, but not one so easily connected to FGM. In the revised version we refrain from referring to FGM when mentioning this hypothesis (lines 520-527).

MK-sensitive substitutions might only be detectable after an environmental shift such that the population needs to chase the optimum and the rate of this is unrelated or not obviously related to population size or to the levels of diversity.

Agreed again. We are not aware of theoretical developments addressing the relationship between number of steps and N_e in an adaptive bout. Some of Orr's papers are close, but none that we could find actually quantifies this relationship. However, two papers have addressed this question by simulating coding sequence evolution under FGM and a moving optimum

(Razetto-Barry et al 2012; Lourenco et al 2013). ω_a is only weakly affected by N_e in these simulations, This is because large populations reach closer to the phenotypic optimum than small populations, but they do not take more adaptive steps in doing so. We briefly recall these results in the revised version (lines 502-218).
