# Supplementary material for: Is adaptation limited by mutation? A timescale-dependent effect of genetic diversity on the adaptive substitution rate in animals
Source: PLoS Genet. 2020 Apr 6;16(4):e1008668. doi: 10.1371/journal.pgen.1008668 (PMC7162527; doi:10.1371/journal.pgen.1008668)
Supplement: Attachment — Submitted filename: Response_to_reviewers.pdf [file pgen.1008668.s016.pdf]

Response to reviewers for the revision of the article untitled "Is adaptation limited by mutation? A timescale-dependent effect of genetic diversity on the adaptive substitution rate in animals", by Rousselle M, Simion P, Tilak MK, Figuet E, Nabholz B, Galtier N. for PloS Genetics

Reviewer #1: The authors have successfully addressed all my comments and corrected most typos. The discussion is richer now than in previous versions and the results are represented and discussed more clearly. This manuscript revisits a fundamental question in population genetics and represents a major stepping stone in our field. I do not have any other major or minor comment (just check for more typos, for example line 452 end of the sentence).

Thank you again for your previous suggestions to improve this manuscript. We corrected the typo at line 452 and screened the manuscript for others.

Reviewer #2: In their manuscript, Rousseau et al. use a large dataset resulting from an impressive sampling effort of coding sequences in very diverse animal species, to provide an updated theory of the differences in adaptation rates between species. Even though the sequencing effort made by the authors is impressive, some of the correlations found by the authors still have limited statistical support. However, the authors convincingly present their results as supporting their proposed model of adaptation rate evolution. The authors are careful to present their results as a working theory that will require more data to validate in the future.

I do not believe that makes this paper less important, quite the opposite actually. This is a landmark paper that will guide efforts in the next five to ten years to understand differences in rates of adaptation between species. It provides at last a broadly explanatory roadmap for what to test to explain different rates of adaptation between different species, beyond the annoyingly simplistic past claims that adaptation just correlates linearly with population size.

In that respect, I share the same views as Adam Eyre Walker about the limitations of the study, but I also strongly believe that these limitations are unavoidable until we have at least an order of magnitude more coding sequence data to explore the proposed model further. This does not decrease the great merit of the manuscript, which is to pave the way for a more comprehensive understanding. Not everything can be 100% certain at first, and believing so represents a gross misunderstanding of the scientific process, that hurts and slows down scientific progress.

I have only a few comments. Castellano and Eyre Walker made great points and have covered a lot of ground already. The reviewers from PCI Evol Biol also covered a lot of ground and I will not repeat their requests as they appear to have been properly taken into account by the authors.

In the methods I could not find the part on the accounting for slightly beneficial mutations that do not fix so fast that they do not contribute to PN. This is potentially important and Galtier 2016 is a

little succinct on how the MK test is robust to slightly beneficial mutations creating an excess of high frequency non-synonymous variants that can bias adaptation rate estimates. The authors need to elaborate more.

We reformulated the M&M section to elaborate on the treatment of weakly beneficial mutations (lines 739 and 748 to 753).

Slightly beneficial mutations that contribute to the SFS are actually taken into account in two of the three models used to build the final estimate of ω_a, i.e. the models GammaExpo and ScaledBeta.

In the GammaExpo model, both a negative Gamma distribution and an exponential distribution are fitted to the SFSs, the exponential distribution modeling the positive part of the DFE. Weakly advantageous mutations that contribute to the high frequency classes of the non-synonymous SFS are thus captured by this exponential distribution.

In the ScaledBeta model, weak-effect mutations (both negative and positive, with S (i.e. $4N_es$) ranging from -25 to 25) are captured by a Beta distribution.

The contribution of these two models, and the third model where no beneficial mutation are considered in the SFS, are then averaged based on their AIC weight, such that the three models contribute to the final estimate according to how well they fit the data of the considered species. So, our approach takes into account between species differences in the contribution of segregating beneficial mutations.

Given recent results on strong purifying selection at synonymous sites in species such as Drosophila, I believe that another complicating factor could be the amount of purifying or positive selection at synonymous sites, and how it varies depending on population size. This can be mentioned succinctly in the Discussion.

Indeed, selection at synonymous sites consists in a violation of the assumptions underlying the DFE-α test, and may thus be a complication in our study. We added a short paragraph discussing how this can influence the among-group relationship between ω_a and π_s in the discussion (see lines 485 to 492).

Minor:

P14-l341: you need to specify which kind of artefact related to fluctuations in population size. I assume that the authors refer more specifically to recently smaller population sizes, that may result in both smaller Pi_s and higher PN/PS and thus lower omega_a?

Recently smaller population sizes might indeed create spurious positive relationship between ω_a and π_s, but we think that the use of the r_i's parameters to correct the SFS relative to the

theoretical neutral expectation might accurately correct for recent fluctuations in population size that have visible consequences in the SFS. At line 341 we refer in the first place to the potential impact of ancient fluctuations that can't be accounted for because they do not impact the shape of the SFS, but impact the dN/dS ratio.These have been showed to potentially yield spurious evidence of positive selection, and possibly a spurious positive correlation between ω_a and π_s (as discussed at lines 389 to 395).

We made clearer in the result section the kind of potential source of bias was investigated via the simulations (see lines 342 to 345).

Reviewer #3: I previously reviewed this manuscript for PLoS Biology and I'm happy that the authors have addressed my concerns and comments. This is a very interesting analysis and I'm happy to recommend acceptance.

Adam Eyre-Walker

Thank you again for your previous comments and suggestions to improve this manuscript.
